# Dynamic rasterstereography improves the detection of movement delays and dynamic asymmetries in the scapulothoracic kinematic of healthy subjects

**DOI:** 10.1002/jeo2.70115

**Published:** 2024-12-18

**Authors:** Richard Julius Freytag, Jonas Wilhelm Moss, Filippo Maria Piana Jacquot, Jakob Zapatka, Rebecca Herrmann, Mahmoud Ragab, Sebastian Scheidt, Davide Cucchi

**Affiliations:** ^1^ Department of Orthopaedics and Trauma Surgery University Hospital Bonn Bonn Germany; ^2^ U.O.C. 1° Clinica Ortopedica ASST Centro Specialistico Ortopedico Traumatologico Gaetano Pini‐CTO Milano Italy

**Keywords:** augmented reality, physiotherapeutic interventions, rasterstereography, reproducibility, scapula, scapular dyskinesis, scapulothoracic kinematics, stereography

## Abstract

**Purpose:**

Assessing scapulothoracic kinematics typically involves visually observing patients during movement, which has limited inter‐ and intraobserver reliability. Dynamic rasterstereography (DRS) records, measures and visualizes surface structures in real time, using a curvature map to colour‐code convex, concave and saddle‐shaped structures on the body surface. This study aimed to evaluate the diagnostic efficacy of DRS‐assisted observation in identifying dyskinetic scapulothoracic patterns.

**Methods:**

Thirty‐seven healthy participants performed shoulder abduction/adduction and flexion/extension cycles without additional weight, recorded using both DRS and a conventional video camera. A metronome ensured consistent timing, and for DRS a grid of parallel light rays projected onto the back surface was captured using indirect optical measurement techniques. The mean surface curvature was converted into a colour scale. The diagnostic performance of conventional and DRS videos in detecting dyskinetic patterns, including static asymmetries, dynamic asymmetries, motion delays and rapid compensatory movements, were compared. Two investigators independently evaluated the videos twice in a blinded and randomized sequence to assess intra‐ and interrater reproducibility.

**Results:**

Analysis of 118 videos showed good‐to‐excellent intrarater and interrater reproducibility for both techniques (ICCs 0.727–0.949). Movement delays and dynamic asymmetries were observed more frequently when evaluating DRS videos rather than conventional videos (*p* = 0.0008 and *p* = 0.0016). However, no differences were found in static asymmetry and rapid compensatory movement detection.

**Conclusions:**

DRS can create a real‐time model of the trunk surface and allows observers to evaluate the scapular movements with good‐to‐excellent intrarater and interrater reproducibility; compared to clinical observation, some specific scapular motion alterations can be observed more frequently.

**Clinical Trial Registration:** Part of the DRKS00022334 trial.

**Level of Evidence:**

Level II, prospective cohort study.

**Abbreviations**
DRS: dynamic rasterstereography
ICC: intraclass correlation coefficient

## INTRODUCTION

The scapula supports upper extremity movement through coordinated muscle activation, enabling six degrees of freedom. Evaluating scapulothoracic kinematics is challenging due to its anatomical and physiological complexity. Various methods have been described to evaluate scapular kinematics, including clinical examination, surface tracking systems, magnetic and inertial sensors, radiographs and computed tomography measurements. In nonresearch settings, visual observation at rest and during active flexion or elevation in the plane of the scapula, with or without palpation of bony landmarks and use of added weights, are the most commonly used methods. These methods rely on an indirect and intuitive observation of muscle activation patterns. While observation‐based methods are noninvasive and avoid unnecessary radiation exposure for the patient, they have limited inter‐ and intraobserver reliability [11, 19, 22, 28]. The main objective of this study was to test the clinical standard against an alternative technique that avoids radiation exposure and skin injury, and could be, therefore, well‐suited for clinical application. Rasterstereography is a noninvasive, light‐optical technology used to record, measure and visualize surface structures, and is well established to monitor the progression of scoliosis, with a documented excellent precision in this field [15, 41, 50]. Recently, there have been applications expanding the scope of this technology to include the evaluation of the shoulder girdle [46]. Dynamic rasterstereography (DRS) can be used to produce and display real‐time images that use a curvature map to colour‐code convex, concave and saddle‐shaped structures on the body surface, thus upgrading optical videos of the patient's back to ‘augmented reality’ videos. This study aims to evaluate the diagnostic performance of DRS‐assisted visual observation in detecting asymmetries and dyskinetic patterns in healthy subjects. The primary goal of this preclinical study was to test the diagnostic efficacy of DRS‐assisted observation in identifying dyskinetic scapulothoracic patterns against the current clinical standard of optical observation and evaluate its reproducibility. We hypothesize that DRS‐assisted visual observation will have superior diagnostic performance and reproducibility compared to standard optical observation.

## MATERIALS AND METHODS

This prospective study (IRB approval: Ethik‐kommission an der Medizinischen Fakultät der Rheinischen Friedrich‐Wilhelms‐Universität Bonn, No. ID 419/19 + Amendment 20.05.2021.) enroled 37 healthy, asymptomatic subjects with unrestricted full range of motion of the shoulder; exclusion criteria were the presence of current or previous pathology of the shoulder girdle, or upper limb and deformities of the trunk or the spine. Subjects were recruited on a voluntary basis with announcements in the centre where the study was performed and in the affiliated university. Dyskinesis, which is commonly observed in asymptomatic individuals, particularly young, active people (prevalence ranging from 8.5% to 92%, and average of 48% in asymptomatic individuals [37, 38, 39]) was not considered an exclusion criterion, in order to better represent the general population.

**Figure 1:**
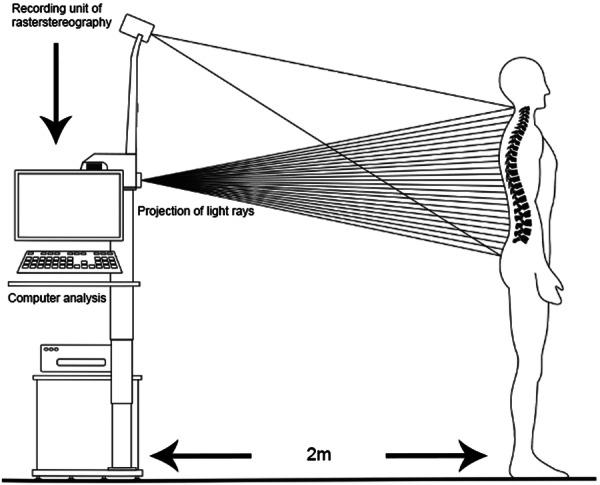
Schematic illustration of the setup featuring the DIERS 4Dmotion®Lab, which generates the rasterstereography recording by projecting a grid of light rays onto the subject's back surface. Subsequently, the recording unit measures the curvatures and the collected data are utilized to generate the dynamic recording. Additionally, a video camera captures a conventional recording for direct comparison purposes. Courtesy of DIERS International GmbH, Schlangenbad, Germany.

Each subject was asked to perform a uniform repetition of 10 cycles of shoulder abduction/adduction and of 10 cycles of shoulder flexion/extension, without additional weight, starting from the neutral position (0° of flexion and 0° of abduction). Each cycle lasted 6 s, divided in two phases: 3 s from the neutral position to reach maximum range of motion (in abduction or flexion) and 3 s to return to the neutral position [25]. A digital metronome was used to create a consistent timing framework. These movements were recorded for each subject by two video cameras (both 60 fps, one for conventional video and one for DRS) placed 2 m behind the subject. This resulted in two/four recordings for each subject being created (conventional, abduction/adduction and/or flexion/extension; DRS, abduction/adduction and/or flexion/extension). Dedicated reflective markers on the scapula are not required for this acquisition (Figure 1).

**Figure 2:**
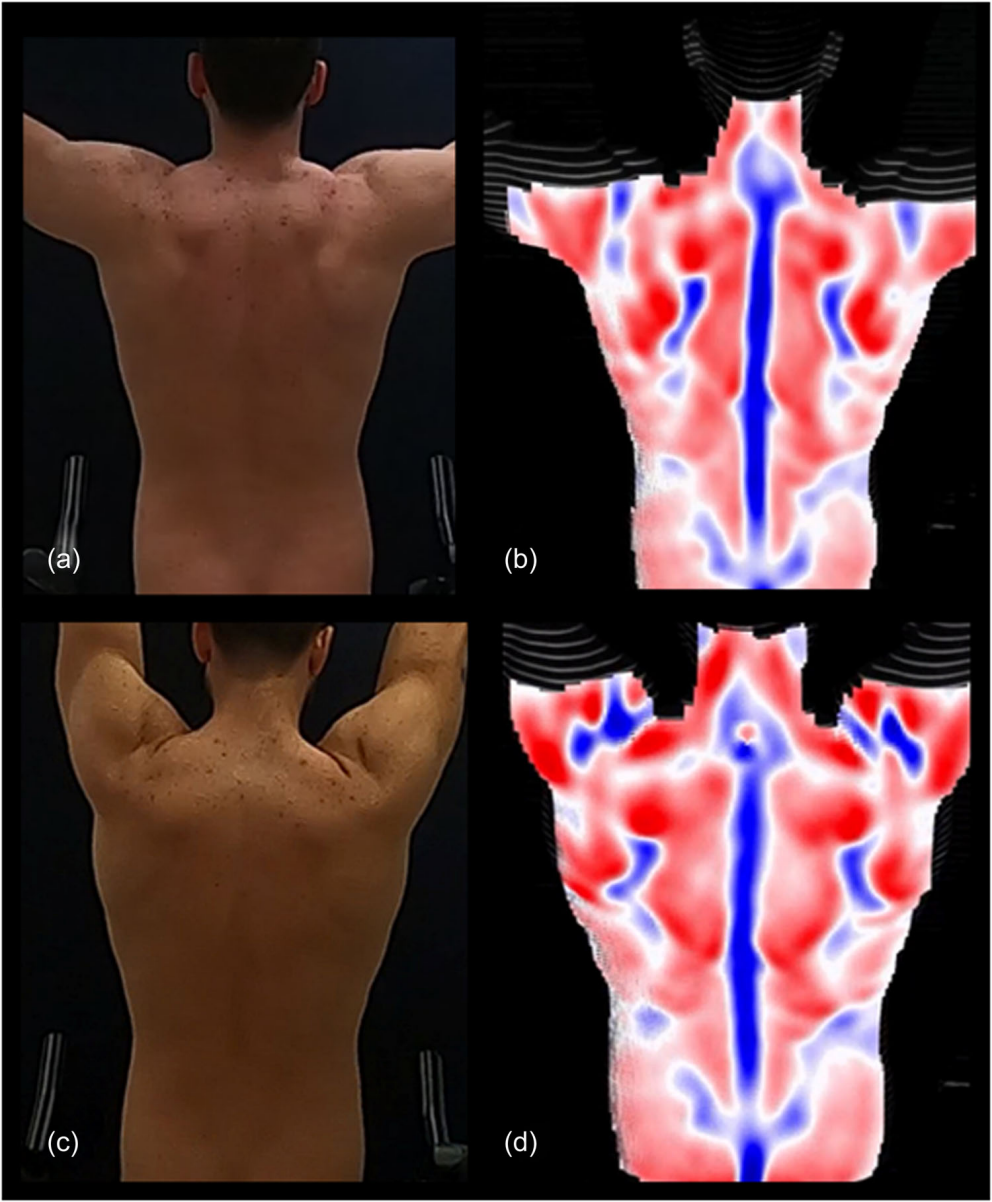
Demonstrative examples of optical and rasterstereography recordings during abduction. (a) Optical image capture of a subject at 90° of abduction. (b) Optical image capture of a subject approaching 180° of abduction. (c) Dynamic rasterstereography (DRS) image capture of a subject at 90° of abduction. (d) DRS image capture of a subject approaching 180° of abduction.

To model the trunk surface during DRS acquisition, a grid of parallel light rays is projected onto the back of the patient from a projector placed at the same distance as a conventional video camera. The light rays are captured using indirect optical measurement methods and corresponding sensors. The DIERS Formetric system (DIERS International GmbH), which is based on rasterstereography technology, projects structured light through a grid pattern to generate a detailed stripe pattern on the patient's body. A Complementary Metal Oxide Semiconductor camera with a recording frequency of 60 Hz captures the curvature of this projected grid. Using triangulation, the associated software (Digital Communication & Application Management, DICAM, v3.11, DIERS International GmbH) computes an individualized surface profile. The mean surface curvature is then colour‐coded, with concave areas shown in blue, convex areas in red and saddle‐shaped areas in white. By correlating the surface profile with the underlying spinal structure, an additional spinal model can be generated [8, 17, 49] (Figure 2).

The collected videos were anonymized by labelling with alphanumeric codes, sorted in a random order to avoid possible bias generated by the sequential observation of videos of the same patient obtained with different techniques and incorporated into an interactive document containing a single recording of a repetition of one subject per page. This preprocessing was performed by an investigator not involved in the subsequent evaluation of the videos (J. M.). Two different observers, both with experience in the diagnosis and treatment of shoulder pathologies including scapulothoracic dyskinesis, evaluated the videos independently (D. C., F. M. P. J.). Prior to study beginning, both observers evaluated and discussed a training set of 12 complete DRS videos of subjects not included in the final cohort, to limit learning curve‐related bias and avoid discrepancies in the interpretation of the study data set. Both raters repeated the evaluation of the randomly sorted videos a second time three months after the first evaluation; this interval was chosen to minimize bias related to the possibility of remembering the rating of the already evaluated videos.

A standardized scheme for the evaluation of the videos was used (Supporting Information S1: Table S1). First, the quality of the video was assessed by evaluating the visibility of six relevant anatomical landmarks (spinal furrow, lumbar fossae, acromion, lateral and medial scapular borders and the inferior angle of the scapula) throughout the entire movement cycle. The evaluation criteria included the discrimination of these landmarks from the surrounding soft tissues, their visibility during the whole movement cycle, the presence of proper lighting or contrast and the absence of artifacts or disturbances. A 4‐point scale was used for this quality assessment, ranging from 0 = poor visualization to 3 = excellent visualization (‘quality assessment’). Second, the frames containing the patient at rest (‘static evaluation’) were evaluated. In these frames, the presence or absence of shoulder height asymmetries, a scoliotic position of the spine and asymmetries of the medial and superior scapular borders, as well as the inferior scapular angle were noted. Finally, the videos of the abduction and flexion cycles were analyzed again (‘dynamic evaluation’) to identify the following: the presence of movement delays, defined as delayed superior rotation and/or delayed tilt of the scapula at 60° and 120° of abduction/flexion; the presence of dynamic asymmetries, defined as alterations in scapulohumeral rhythm, posterior tilt of the inferior scapular angle, posterior and superior tilt of the medial scapular border, compensatory shoulder elevation, scapular winging or asymmetric prominences of the medial, superior and inferomedial scapular border in any phase of the cycle and the presence of ‘rapid compensatory movements’, defined as swift, abnormal and sudden movement of one scapula compared to the contralateral side during the flexion/abduction cycle. These movements may manifest in either a cranio‐caudal direction (superior border rapid movement) or a medio‐lateral direction (medial border rapid movement). The presence of rapid compensatory movements was documented along with the phase of the cycle in which the movements were detected: a full cycle was segmented into ascending and descending phases, and each of these phases was further divided into specific ranges of movement (0–60°; 60–120°; 120–180°). Both shoulder girdles were evaluated separately in each video. Finally, the observer summarized the presence of any asymmetries and was required to indicate if scapulothoracic dyskinesis was present. The checklist used for the standardized evaluation of the scapulothoracic kinematics is available in the supplementary materials (Supporting Information S1: Table S1).

Statistical analysis was performed using GraphPad Prism v 6.0 software (GraphPad Software Inc.) and Microsoft Excel (Microsoft Corporation). The normality of the sample distribution was assessed using the Shapiro–Wilk test. Continuous variables were presented as either mean ± standard deviation or as medians with the first and third quartiles (Q1–Q3), depending on the data distribution. Interobserver agreement was determined using the intraclass correlation coefficient (ICC) for the measured parameters: ICC values below 0.4 indicated poor reliability, values between 0.4 and 0.75 indicated good reliability, and values above 0.75 indicated excellent reliability [9]. Differences in continuous variables were assessed using either an unpaired Student's *t* test or Mann–Whitney test, depending on the distribution of the data. Categorical variables were expressed in numbers of cases and frequencies; their differences were tested using the *χ* ^2^ test or Fisher's exact test. A significance level of *p* < 0.05 was considered statistically significant for all analyses. Sample size calculation was based on a preliminary analysis (*n* = 10), which revealed dynamic asymmetries in 50% of conventional videos and in 85% of DRS‐assisted recordings of the same subjects. Based on these data, and assuming a power level of 80% and a significance level of 5% for a two‐sided comparison, the minimum sample size required for investigation was determined to be 50 movement acquisitions (resulting in 100 videos, as each was recorded using both conventional video and DRS).

## RESULTS

In this study, 60 pairs of videos were collected and 118 videos were analyzed (60 optic and 58 DRS‐augmented, 70 depicting abduction and 48 flexion cycles). The mean age of the study population was 29.13 ± 5.61 (females: 67/118, 57%; right‐hand dominance: 100/118, 85%).

The intrarater and interrater reproducibility for evaluating the presence of asymmetries and dyskinesis was deemed good to excellent for both techniques (ICC: 0.727–0.949). Similar ICCs were documented in all subgroup comparisons, with the exception of a lower interrater consistency that was observed for the DRS‐augmented videos during flexion compared to that measured for videos in abduction (Supporting Information S2: Table S2).

**Figure 3:**
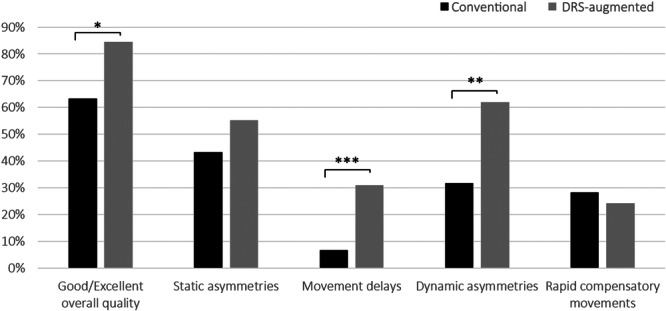
Bar charts comparing different study outcomes between conventional and dynamic rasterstereography‐augmented videos. The y‐axis indicates the study outcome frequency. ***p < 0.001; **p < 0.01; *p < 0.05.

When comparing observations from the same subject performing identical abduction/adduction or flexion/extension movement cycles recorded with both techniques, we found that DRS provided improved visibility of key anatomical landmarks in 56.9% of cases, with no difference noted in 8.6%; overall video quality was rated higher with DRS in 43.1% of cases, with no difference in 31%. In 29.3% of cases, DRS detected movement delays not visible in the corresponding conventional videos (same detection rates in 67.2% of the cases) and in 43.1% of cases more dynamic asymmetry were detected with DRS (no difference in 37.9% of the cases). Regarding the detection of static asymmetries and rapid compensatory movements, most individual assessments yielded the same results with both the conventional recording and DRS (46.6% and 70.7%, respectively). As a result, DRS significantly outperformed conventional videos in visualizing anatomical landmarks (*p* = 0.0119) and detecting movement delays (*p* = 0.0008) and dynamic asymmetries (*p* = 0.0016). No differences were found in static asymmetry and rapid compensatory movement detection (Figure 3). Similar results were partially observed in subgroup analyses of subjects with and without scapulothoracic dyskinesis (Supporting Information S3: Table S3).

**Figure 4:**
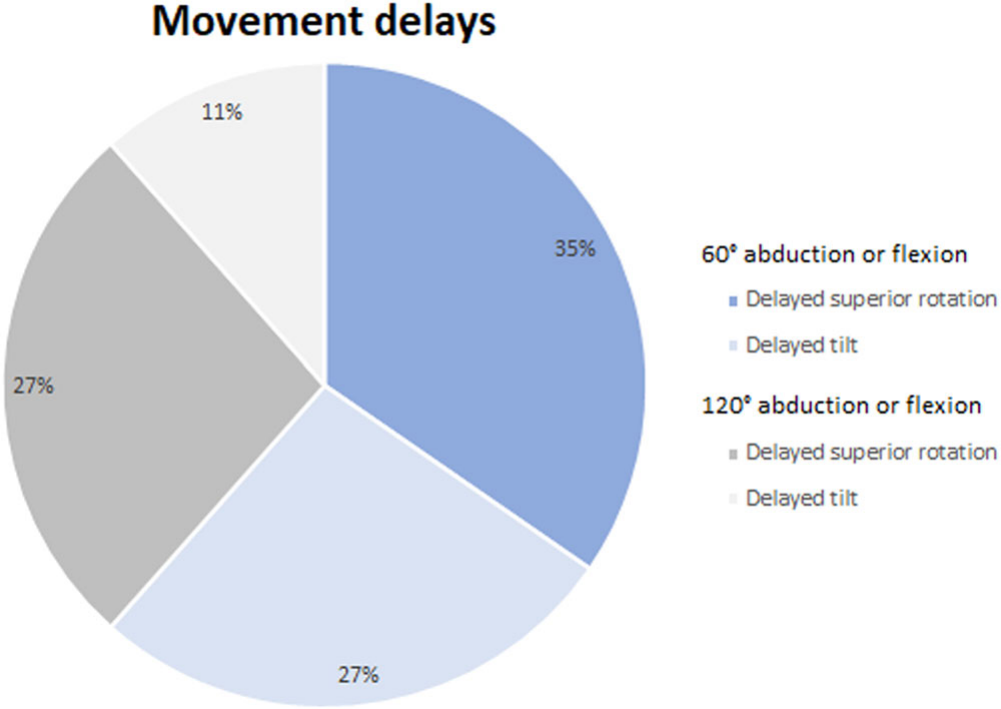
Pie charts illustrating the frequency of movement delays detected during two different moments of the scapular motion cycle.

The standardized assessment tool used enabled to explore the nature of the encountered asymmetries in the scapulothoracic movements in detail. Movement delays involved predominantly the scapular upward rotation and were observed most frequently at the end of the first third of the cycle (60° abduction or flexion, Figure 4).

**Figure 5:**
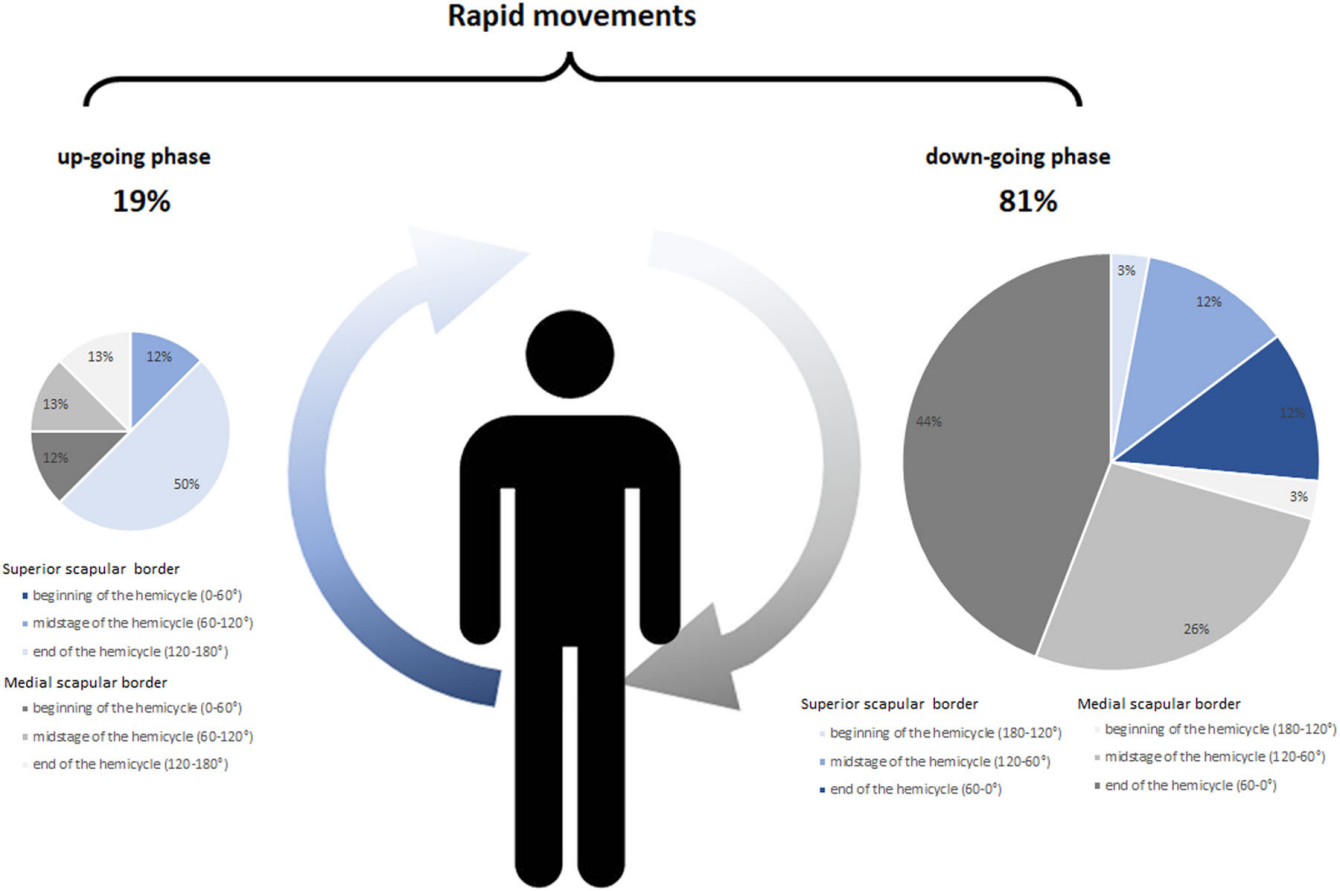
Schematic illustration and pie charts depicting the frequency of rapid movements during the different phases of the scapular motion cycle.

Rapid movements were detected with both investigation techniques throughout the scapular abduction and flexion cycles, nevertheless, their frequency was four times higher in the down‐going phase as in the up‐going phase; the most frequently observed compensatory movements were rapid movements of the medial scapular border during the end of the down‐going phase (Figure 5).

Dyskinesis frequency was 20% in the conventional and 22% in the DRS‐assisted observations.

## DISCUSSION

The main finding of this study is that DRS identified movement delays and dynamic asymmetries during shoulder movement more frequently as compared to conventional observation. This digital technology, which can create a real‐time dynamic model of the surface of the trunk, shows good intra‐ and interrater reliability and appears, therefore, promising for enhancing scapular kinematics and dyskinesis assessment in clinical and research settings.

In a physiological condition, control of static position and dynamic motion is accomplished by patterned muscle activations that place the scapula in optimal position between the stable base of the trunk and the mobile arm [27]. Dyskinesis is a nonspecific response to a pathological or painful condition at the shoulder level, scapular girdle, trunk or neck or alterations in posture [14, 20, 44]. While dyskinesis often suggests a deviation from normal scapular motion, it is commonly observed even in asymptomatic individuals, particularly young, active people. Prevalence in these populations varies widely, from 8.5% to 92%, with a recent study by Salamh et al. showing an average prevalence of 48% in the general asymptomatic population [37, 38, 39]. Our study detected a lower prevalence of dyskinesis, consistent with that reported in previous publications focused on young, active individuals who are not professional overhead athletes [5, 18, 42]. Dyskinesis, although frequently asymptomatic, may still affect long‐term athletic performance, with Hickey et al. noting a 43% increased risk of shoulder pain among athletes with dyskinesis [16]. Thus, scapulothoracic assessment is relevant not only in posttrauma or postsurgical rehabilitation but also in assessing athletic performance. In these contexts, experimental reference methods—such as tracking with bony pins, electromagnetic or optical devices or radiological imaging—may be impractical due to time, cost or invasiveness. Three‐dimensional surface tracking systems are the reference standard for scapulothoracic kinematic evaluation in research settings; [31, 47] however, they show systematic errors in assessing complex shoulder movements, and their accuracy relies on skin and tissue characteristics and precise landmark identification. Additionally, patient preparation is time‐consuming and requires skilled personnel, limiting their clinical use [12, 24, 29, 43]. Radiological techniques allow the recognition of bony structures with excellent definition, albeit at the cost of exposure to ionizing radiation [4, 6, 32, 33, 34, 35, 36]. Inertial sensors and electromyography are highly promising but currently mainly confined to research environments due to the high complexity of patient preparation and/or data acquisition [1, 23, 48, 51]. Therefore, outside of research contexts, observation of the trunk and scapulae at rest and during active abduction or flexion, with or without palpation of bony landmarks, remain the most commonly used methods, currently accepted as clinical gold standard. These noninvasive methods, based on indirect observation of muscle activation patterns and bone displacement, have limited inter‐ and intraobserver reliability, mainly because beyond obvious dyskinesis, more subtle asymmetries may exist, such as minor differences in muscle activation patterns or timing, which may variably alter the raters' judgement thus decreasing reliability of classifications [19, 22, 28, 43]. The primary aim of this study was to challenge this clinical standard against an alternative technique that requires no additional time, does not expose patients to radiation, and avoids skin injuries, making it feasible for clinical use. Rasterstereography is a noninvasive optical technology used to acquire, measure and visualize surfaces, based on the principle of triangulation combined with image processing techniques. Since the 1980s, this technique has been applied to visualize the posterior trunk surface, aiding in lumbar and thoracic spine studies, reducing the cumulative need for X‐rays and allowing reliable measurement of parameters related to coronal and sagittal alignment and spinopelvic torsion [13, 15, 26, 41, 50]. Beyond scoliosis, rasterstereography has been used to study leg length discrepancy's impact on pelvic position and compensation mechanisms in both healthy subjects and hip prosthesis patients [2, 3]. Its accuracy and reproducibility have been studied in both spinal deformity and lower limb alignment assessments, confirming the technique as reliable [21, 26, 30, 45]. Rasterstereography found its first application in the study of the scapular girdle in the context of juvenile idiopathic scoliosis, in a study which aimed to assess the reliability and validity of the technique to measure static parameters related to shoulder position [46]. As compared to initial application with only static images (single or averages of image acquisitions with the subject in the same position), today longer rasterstereographic acquisitions can be performed under dynamic conditions (DRS) [7, 10, 50]. By representing the measured curvatures at each point throughout the acquisition with a predefined colour coding, a video is obtained that can enhance the conventional optical visualization of the trunk and scapulae in motion, ‘augmenting’ the observed reality. This technology is of particular interest because it is a noninvasive and radiation‐free procedure that does not require the use of specially trained personnel [40]. Potential future applications range from the follow‐up of patients to athletic preparation to automatic, artificial intelligence‐based rasterstereographic evaluation.

In the preliminary experience presented in this study on healthy asymptomatic subjects, DRS increased the detection frequency of movement delays and dynamic asymmetries: the authors attribute this to a simpler and yet more accurate assessment of discrepancies which is done comparing the distribution and dimensions of areas in blue, red and white, in contrast to optical evaluation, where the only indicators are shadows and skin folds. In the authors' subjective experience, this technology proved particularly advantageous for evaluating overweight subjects (helping overcome limitations imposed by morphotypes in optical assessments). Furthermore, the use of a checklist for the standardized evaluation of the scapulothoracic kinematics proved to be of advantage, appearing to also increase the reliability of conventional observation methods [12, 19, 22].

This study has some limitations: first of all, this was a pilot study on healthy volunteers without any active or previous pathology of the trunk, spine shoulder gridle and upper limb; therefore, the authors suggest care when transferring these results to clinical practice. Asymptomatic volunteers were included, yet without excluding those with asymptomatic scapulothoracic dyskinesis, which can add some heterogeneity in the study population; nevertheless, this approach offers a more representative view of the general population, given the rarity of complete absence of scapular asymmetry [38]. Although the chosen evaluation method was highly standardized, some bias related to subjectivity in the interpretation of complex movement patterns and potential human error may still persist. Additionally, fully automated DRS analysis is not yet available, which could further minimize such biases in the future. The lack of an experimental gold standard, such as bony pin tracking, electromagnetic devices or radiological imaging, is a further limitation of this study. However, visual observation during scapular movement is the clinical gold standard in practice, and this study aimed to evaluate it against an alternative approach that is similarly time‐efficient, avoids radiation and is feasible for clinical use. Finally, the currently high associated acquisition costs and the need for some dedicated software updates for the analysis of the scapulothoracic kinematic can pose a substantial challenge: this leads to a scarcity of centres equipped with this technology, which hinders large‐scale data collection, essential for confirming its reliability.

Several technical aspects must be considered when implementing a DRS setting to avoid pitfalls during clinical care. First, ensuring the solidity of the support for the conventional video camera is crucial, as minor tilts in the acquisition may necessitate postproduction editing. Second, sufficient data storage, synchronization capacity and working memory needs to be planned to ensure seamless acquisition and processing. DRS clips generated are typically large (2GB/recording) and slower than real‐life speed, requiring dedicated speed‐matching and compression for visualization on standard computers. Finally, the ideal movement sequence and number of repetitions have not been investigated in detail yet; the results of this study show better interrater reliability in the evaluation of abduction cycles and a reduced number of movement cycles could be considered in future applications to optimize time efficiency and limit muscular fatigue.

## CONCLUSIONS

DRS can generate a real‐time dynamic model of the trunk surface, facilitating the evaluation of scapulothoracic kinematics in a straightforward manner and with good‐to‐excellent intrarater and interrater reproducibility. Compared to conventional observation methods, some specific scapular motion alterations can be observed more frequently. These attributes render this technique promising in clinical settings as possible adjunct in the assessment of scapular kinematics.

## AUTHOR CONTRIBUTIONS

**Richard Julius Freytag**: Study design; manuscript correction. **Jonas Wilhelm Moss**: Data collection; original draft preparation; figures. **Filippo Maria Piana Jacquot**: Data analysis original draft preparation. **Jakob Zapatka**: Data collection; figures. **Mahmoud Ragab**: Data collection. **Rebecca Herrmann**: Data collection. **Sebastian Scheidt**: Manuscript correction. **Davide Cucchi**: Study design; data analysis original draft preparation.

## CONFLICT OF INTEREST STATEMENT

Author S. S. declares travel/accommodation reimbursements from Arthrex outside the presented work. Author D. C. declares travel/accommodation reimbursements from Lima outside the presented work. The remaining authors declare no conflict of interest.

## ETHICS STATEMENT

Institutional review board approval number: Ethics Committee of the Medical Faculty, University Hospital Bonn, University of Bonn, Building 74/4th floor, Venusberg‐Campus 1, 53105 Bonn, Germany, No. ID 419/19. Informed consent was obtained from all individual participants included in the study. Permission to reproduce material from other sources was obtained for Figure 1.

## Supporting information

Supporting information.

Supporting information.

Supporting information.

Supporting information.

## Data Availability

The data that support the findings of this study are available on request from the corresponding author. The data are not publicly available due to privacy or ethical restrictions.
