# Supplementary material for: Dynamic rasterstereography improves the detection of movement delays and dynamic asymmetries in the scapulothoracic kinematic of healthy subjects
Source: J Exp Orthop. 2024 Dec 18;11(4):e70115. doi: 10.1002/jeo2.70115 (PMC11653215; doi:10.1002/jeo2.70115)
Supplement: Supplementary file 1 — Supporting information. [file JEO2-11-e70115-s003.pdf]

		LEFT SHOULDER	RIGHT SHOULDER
**Video quality - for each item visibility: 3 (Excellent); 2(Good); 1 (Fair); 0 (Poor**			
Spinal furrow ("back valley")			
Fossae lumbares			
Acromion			
Lateral border of the scapula			
Medial border of the scapula			
Inferior angle of the scapula			
Overall quality of the video			
**Static Evaluation**<br>**position before the video starts - 0° flexion/abduction**	**for each item: 0 (absent); 1 (present)**		
Asymmetry of the shoulder height			
Scoliotic position of the spine			
Asymmetry of the medial scapular border			
Asymmetry of the superior scapular border			
Asymmetry of the inferior scapular angle			
**Dynamic Evaluation**<br>**position before the video starts - 0° flexion/abduction**	**for each item: 0 (absent); 1 (present)**		
**At 60° of flexion/abduction**			
Delayed superior rotation			
Delayed tilt			
**At 120° of flexion/abduction**			
Delayed superior rotation			
Delayed tilt			
**In any point of the flexion/abduction cycle**			
Alterations in scapulohumeral rhythm			
Dorsal tilt of the inferior scapular angle			
Dorsal and superior tilt of the medial scapular border			
Compensatory shoulder elevation			
Scapula winging			
Asymmetric prominence of the	medial scapular border		
	superior scapular border		
	Inferomedial scapular border		
**Presence of rapid movement in the up-going phase**			
Superior scapular border	beginning of the cycle (0-60°)		
	midstage of the cycle (60-120°)		
	end of the cycle (120-180°)		
Medial scapular border	beginning of the cycle (0-60°)		
	midstage of the cycle (60-120°)		
	end of the cycle (120-180°)		
**Presence of rapid movement in the down-going phase**			
Superior scapular border	beginning of the cycle (0-60°)		
	midstage of the cycle (60-120°)		
	end of the cycle (120-180°)		
Medial scapular border	beginning of the cycle (0-60°)		
	midstage of the cycle (60-120°)		
	end of the cycle (120-180°)		
**Overall Evaluation: Dyskinesis**	**0 (absent); 1 (present)**		
Dyskinesis			

Checklist for the standardized evaluation of the scapulothoracic kinematics
Freytag RJ, Moss J, Piana Jacquot FM, Zapatka J, Herrmann E, Ragab R, Sebastian R, Cucchi D
Universitätsklinikum Bonn, Klinik und Poliklinik für Orthopädie und Unfallchirurgie.

schulter@ukbonn.de

UNIVERSITÄT BONN

ukb universitäts klinikumbonn
