# Supplementary material for: Dynamic rasterstereography improves the detection of movement delays and dynamic asymmetries in the scapulothoracic kinematic of healthy subjects
Source: J Exp Orthop. 2024 Dec 18;11(4):e70115. doi: 10.1002/jeo2.70115 (PMC11653215; doi:10.1002/jeo2.70115)
Supplement: Supplementary file 2 — Supporting information. [file JEO2-11-e70115-s001.docx]

Supplementary Table S2: detailed overview of intra-rater and inter-rater reproducibility of the evaluation of the presence of asymmetries and dyskinesis, including subgroup analysis based on the direction of the movement (abduction and flexion) and the image acquisition technique (optic and DRS-augmented).

|  | | Overall | | Subgroup analysis: abduction and flexion videos | | | | Subgroup analysis: optic and DRS-augmented videos | | | | *Subgroup analysis: abduction and flexion, optic and DRS-augmented videos* | | | | | | | |
| --- | --- | --- | --- | --- | --- | --- | --- | --- | --- | --- | --- | --- | --- | --- | --- | --- | --- | --- | --- |
|  |  | ICC | CI | ICC_Abd_ | CI | ICC_Flex_ | CI | ICC_Opt_ | CI | ICC_DRS_ | CI | *ICC_Abd_Opt_* | *CI* | *ICC_Abd_DRS_* | *CI* | *ICC_Flex_Opt_* | *CI* | *ICC_Flex_DRS_* | *CI* |
| Asymmetries | Intra-rater R1 | 0.898 | 0.818 to 0.977 | 0.855 | 0.735 to 0.976 | 0.958 | 0.878 to 1.000 | 0.9 | 0.790 to 1.000 | 0.926 | 0.825 to 1.000 | *0.829* | *0.647 to 1.000* | *0.866* | *0.688 to 1.000* | *1* | *1.000 to 1.000* | *0.917* | *0.757 to 1.000* |
|  | Intra-rater R2 | 0.911 | 0.835 to 0.987 | 0.970 | 0.912 to 1.000 | 0.826 | 0.662 to 0.989 | 1 | 1.000 to 1.000 | 0.770 | 0.579 to 0.961 | *1* | *1.000 to 1.000* | *0.915* | *0.750 to 1.000* | *1* | *1.000 to 1.000* | *0.597* | *0.242 to 0.951* |
|  | Inter-rater | 0.844 | 0.746 to 0.941 | 0.881 | 0.768 to 0.994 | 0.792 | 0.623 to 0.961 | 0.933 | 0.843 to 1.000 | 0.727 | 0.544 to 0.909 | *0.837* | *0.661 to 1.000* | *0.85* | *0.650 to 1.000* | *1* | *1.000 to 1.000* | *0.583* | *0.288 to 0.879* |
| Dyskinesis | Intra-rater R1 | 0.852 | 0.737 to 0.967 | 0.856 | 0.718 to 0.993 | 0.833 | 0.610 to 1.000 | 0.896 | 0.754 to 1.000 | 0.812 | 0.636 to 0.988 | *0.923* | *0.775 to 1.000* | *0.794* | *0.572 to 1.000* | *0.833* | *0.518 to 1.000* | *0.833* | *0.518 to 1.000* |
|  | Intra-rater R2 | 0.875 | 0.478 to 0.982 | 0.897 | 0.782 to 1.000 | 0.777 | 0.479 to 1.000 | 0.857 | 0.699 to 1.000 | 0.895 | 0.752 to 1.000 | *0.929* | *0.791 to 1.000* | *0.866* | *0.685 to 1.000* | *0.700* | *0.311 to 1.000* | *1* | *1.000 to 1.000* |
|  | Inter-rater | 0.848 | 0.730 to 0.966 | 0.885 | 0.757 to 1.000 | 0.692 | 0.366 to 1.000 | 0.949 | 0.851 to 1.000 | 0.745 | 0.534 to 0.956 | *1* | *1.000 to 1.000* | *0.794* | *0.572 to 1.000* | *0.833* | *0.518 to 1.000* | *0.467* | *-0.132 to 1.000* |

CI: confidence interval; ICC: intraclass correlation coefficient; Abd: Abduction; Flex: Flexion; Opt: acquisition with conventional optic camera; DRS: dynamix rasterstereography-augmented acquisition; R1: first rater; R2: second rater.

­
