# Supplementary material for: Dynamic rasterstereography improves the detection of movement delays and dynamic asymmetries in the scapulothoracic kinematic of healthy subjects
Source: J Exp Orthop. 2024 Dec 18;11(4):e70115. doi: 10.1002/jeo2.70115 (PMC11653215; doi:10.1002/jeo2.70115)
Supplement: Supplementary file 3 — Supporting information. [file JEO2-11-e70115-s002.docx]

Supplementary Table S3: Summary of the results of the Fisher’s exact test for categorical data applied to compare different study outcomes between conventional and DRS-augmented videos, within the subgroups of subjects with and without scapulothoracic dyskinesis. Significant values are reported in bold.

|  | No dyskinesis | Dyskinesis |
| --- | --- | --- |
| Good/Excellent overall quality | ***0.0051*** | 1.0000 |
| Static asymmetries | 0.2944 | 0.5930 |
| Movement delays | ***0.0125*** | ***0.0414*** |
| Dynamic asymmetries | ***0.0001*** | 1.0000 |
| Rapid compensatory movements | 0.7411 | 0.5930 |
