# Supplementary material for: Dynamic rasterstereography improves the detection of movement delays and dynamic asymmetries in the scapulothoracic kinematic of healthy subjects
Source: J Exp Orthop. 2024 Dec 18;11(4):e70115. doi: 10.1002/jeo2.70115 (PMC11653215; doi:10.1002/jeo2.70115)
Supplement: Supplementary file 4 — Supporting information. [file JEO2-11-e70115-s004.docx]

Supplementary Table S1: checklist used for the standardized evaluation of the scapulothoracic kinematics.

Supplementary Table S2: detailed overview of intra-rater and inter-rater reproducibility of the evaluation of the presence of asymmetries and dyskinesis, including subgroup analysis based on the direction of the movement (abduction and flexion) and the image acquisition technique (optic and DRS-augmented).

Supplementary Table S3: Summary of the results of the Fisher’s exact test for categorical data applied to compare different study outcomes between conventional and DRS-augmented videos, within the subgroups of subjects with and without scapulothoracic dyskinesis. Significant values are reported in ***bold***.
